# Family resilience and self-management in stroke survivors: a longitudinal cross-lagged analysis

**DOI:** 10.3389/fpsyg.2026.1855568

**Published:** 2026-07-09

**Authors:** Linyi Wei, Meng Yang, Li Ma, Xinglei Wang, Hui Yan, Xiaohong Ma, Dou Bai, Xinman Dou

**Affiliations:** 1 School of Nursing, Lanzhou University, Lanzhou, Gansu, China; 2 Department of Nursing, Lanzhou Sanaitang Hospital, Lanzhou, Gansu, China; 3 Department of Surgical Oncology, Lanzhou University Second Hospital, Lanzhou, Gansu, China; 4 Department of Cardiology, Lanzhou University Second Hospital, Lanzhou, Gansu, China; 5 Department of Nursing, Lanzhou University Second Hospital, Lanzhou, Gansu, China; 6 Department of Neurology, Lanzhou University Second Hospital, Lanzhou, Gansu, China

**Keywords:** cross-lagged analysis, family resilience, longitudinal study, rehabilitation, self-management, stroke

## Abstract

**Objective:**

To explore the longitudinal temporal relationship between family resilience and self-management behaviors in stroke patients.

**Methods:**

A longitudinal observational study was conducted with three time points. Family resilience and self-management were assessed at hospitalization (T0), 3 months (T1), and 6 months (T2) post-discharge in 206 stroke patients using validated Chinese scales. A cross-lagged panel model was applied.

**Results:**

Self-management initially increased then declined; family resilience initially increased then stabilized. Self-management at T0 positively predicted family resilience at T1 (*β* = 0.203, *p* < 0.001). Family resilience at T1 positively predicted self-management at T2 (*β* = 0.269, *p* < 0.001).

**Conclusion:**

A time-ordered interactive relationship exists. In early rehabilitation, fostering self-management promotes family resilience; in later stages, enhancing family resilience helps maintain self-management.

## 1 Background

Stroke is a cerebrovascular disease characterized with high incidence and disability rates (National Health Commission of the People's Republic of China, 2025). Its global disease burden is increasingly significant. According to a report from the World Stroke Organization, between 1990 and 2021, the global number of stroke-related deaths increased by 44%, and the prevalence rate increased by 86% (Feigin et al., 2025). In China, the disease burden is particularly severe. A nine-year study revealed that the recurrence rate among first-time stroke patients in China is 17% within one year and reaches 41% within five years (Chen et al., 2020). Meanwhile, approximately 70–80% of survivors still suffer from long-term impairments in cognition, motor function, and self-care abilities, significantly compromising their quality of life (Guo et al., 2024). Due to limited hospital rehabilitation resources, the home has become the primary setting for recovery (Tchero et al., 2018). Thus, effective self-management by patients is crucial for improving long-term prognosis. Self-management is defined as the active participation of an individual in managing his or her physical, emotional, and role-related needs, in order to live well with a chronic condition (Lorig and Holman, 2003). Furthermore, research from the American Heart Association highlights that improving patients’ self-management levels can significantly reduce the risk of stroke recurrence and enhance their quality of life (Riegel et al., 2017).

Among the various factors influencing patient self-management behaviors, family resilience plays a crucial role. Family resilience refers to the capacity of the family, as a functional unit, to effectively withstand and recover from adversity and stress (Tan et al., 2024). Studies have shown that family resilience is a significant positive predictor of self-management behaviors in patients with chronic wounds (Qiu et al., 2024) and adolescents with diabetes (Luo et al., 2019). For stroke survivors, high family resilience fosters a stable rehabilitation environment, provides sustained emotional support, and enables adaptive adjustments to family roles and functioning, thereby offering indispensable external support and a foundation of confidence for patients to engage in self-management behaviors (Zhang et al., 2022).

However, most studies on family resilience and self-management use cross-sectional designs. These cannot determine if a temporal relationship exists or show when one predicts the other. To address this limitation, our study uses a longitudinal design and cross-lagged analysis to thoroughly examine the reciprocal predictive relationships between family resilience and self-management behaviors in stroke patients. The aim is to provide a theoretical foundation for healthcare professionals to develop targeted intervention strategies, ultimately improving patients’ quality of life.

## 2 Participants and methods

### 2.1 Participants

From November 2023 to June 2024, a convenience sampling method was employed to recruit stroke patients from two tertiary hospitals in Lanzhou City as the study participants. The inclusion criteria were as follows: ① meeting the diagnostic criteria outlined in the Cerebrovascular Disease Prevention and Treatment Guide (2024 Edition) (National Health Commission of the People's Republic of China, 2025), with confirmation by CT or MRI imaging; ② being in a stable condition post-treatment, conscious and clear-minded, and able to complete the questionnaires independently or with researcher guidance; and ③ voluntarily agreeing to participate in the study and providing informed consent. The exclusion criteria included: ① having other concurrent life-threatening conditions (e.g., end-stage malignant tumors, severe hepatic or renal failure); and ② having a history of hearing or visual impairment or mental disorders. This study was approved by the Biomedical Ethics Committee of Lanzhou University, (The approval number is: HLLL-20240117).

### 2.2 Survey instruments

#### 2.2.1 General information questionnaire

A self-designed general information form was used to collect data, including age, gender, educational level, occupation, marital status, monthly household income, residential area, living arrangement, number of stroke episodes, family history of stroke, comorbidities, and Rankin score.

#### 2.2.2 The Chinese version of family resilience assessment scale (FRAS-C)

The FRAS-C was translated and adapted into Chinese by Li et al. (2016) in 2016. This scale comprises 32 items across three dimensions: family communication and problem solving, utilizing social resources, and maintaining a positive outlook. It employs a 4-point Likert scale, ranging from 1 (strongly disagree) to 4 (strongly agree). The total score ranges from 32 to 128, with higher scores indicating higher levels of family resilience. In this study, the Cronbach’s *α* coefficient for the FRAS-C was 0.971.

#### 2.2.3 Stroke self-management behavior scale (SSBS)

The SSBS was developed by Wang (2012) in 2012. This 51-item scale assesses seven dimensions of self-management: disease management, safe medication management, diet management, daily life management, emotional management, social function and interpersonal management, and rehabilitation exercise management. Responses are recorded on a 5-point Likert scale from A (1 point) to E (5 points). The total score ranges from 50 to 255, with higher scores reflecting better self-management behaviors. In the present study, the SSBS demonstrated a Cronbach’s *α* coefficient of 0.835.

### 2.3 Data collection

This study enrolled patients who received medical care between November 2023 and February 2024. Data collection occurred at three time points: during hospitalization (T0), at three months post-discharge (T1), and at six months post-discharge (T2). At T0, the General Information Questionnaire, the Chinese Version of the Family Resilience Assessment Scale (FRAS-C), and the Stroke Self-management Behavior Scale (SSBS) were administered on-site. At T1 and T2, follow-up assessments of family resilience and self-management behaviors were conducted via telephone, WeChat, or face-to-face outpatient interviews.

Upon completion, all questionnaires were immediately checked for omissions. Questionnaires with missing data were excluded. Data were independently entered by two individuals to ensure accuracy.

A total of 219 valid questionnaires were collected at T0. From this baseline cohort, the first follow-up at T1 successfully obtained 217 valid responses (2 participants lost to follow-up). Subsequently, the second follow-up at T2 successfully obtained 206 valid responses from the 217 T1 participants (11 additional participants lost to follow-up). The overall attrition rate for this study was 5.9%. Primary reasons for attrition included inability to contact participants, refusal to continue in the study, and death.

### 2.4 Statistical analysis

Data were analyzed using SPSS version 27.0. Normally distributed continuous data are presented as the mean ± standard deviation, while non-normally distributed continuous data are presented as the median (interquartile range). Missing data were handled using listwise deletion (i.e., only participants with complete data at all three time points were retained). Harman’s single-factor test was employed to assess common method bias, with a variance contribution rate exceeding 40% by the first factor indicating severe bias. The relationship between family resilience and self-management behaviors in stroke patients was examined using Spearman’s correlation analysis. A cross-lagged model was constructed using Mplus version 8.0 to explore the predictive relationship between family resilience and self-management behaviors. The model fit was evaluated using the following indices: χ^2^/df < 5, CFI > 0.90, TLI > 0.90, RMSEA < 0.08, and SRMR < 0.08. The significance level was set at *α* = 0.05. No covariates were included in the cross-lagged model due to sample size limitations.

## 3 Results

### 3.1 General characteristics of the stroke patients

This study initially enrolled 219 stroke patients, of whom 206 completed the entire follow-up process. No statistically significant differences were found in the general characteristics between the participants who completed the study and those who were lost to follow-up or withdrew.

Among the total 219 enrolled patients, 163 were male and 56 were female. Regarding stroke type, 56 cases were hemorrhagic, while 153 were ischemic. The age distribution was as follows: 4 patients were under 45 years old, 19 were between 45 and 60 years old, and 196 were over 60 years old. In terms of educational attainment, the majority (125 participants) had a high school or vocational secondary school education. Most participants (173) lived in non-solitary arrangements, and family members served as the primary caregivers for the vast majority (194 participants). The mean time from stroke diagnosis to the T0 assessment (during hospitalization) was 7.51 years (SD = 9.02 years, range: 0.10–48.00 years), based on the 219 enrolled patients. For detailed information, please refer to Table 1.

**Table 1:** Comparison of sociodemographic characteristics between patients who completed follow-up and those lost to follow-up (*n* (%)).

Variable	Category	Completed follow-up group (*n* = 206)	Lost-to-follow-up group (*n* = 13)	*p*
Age				0.100
	18 ~ 44	4 (1.9)	0 (0.0)	
	45 ~ 59	11 (5.3)	6 (46. 1)	
	60 ~ 74	117 (56.8)	5 (38.5)	
	75 ~ 89	74 (35.9)	2 (15.4)	
Gender	Male	153 (74.3)	10 (76.9)	0.832
	Female	53 (25.7)	3 (23. 1)	
Occupation				0.064
	Employed	14 (6.8)	1 (7.7)	
	Retired	139 (67.5)	6 (46. 1)	
	Worker/Farmer	30 (14.6)	6 (46.2)	
	Freelancer	15 (7.3)	0 (0.0)	
	Unemployed	8 (3.9)	0 (0.0)	
Marital status	Married	160 (77.7)	13 (100.0)	0.075
	Unmarried/Divorced/Widowed	46 (22.3)	0 (0.0)	
Living arrangement	Not living alone	161 (78.2)	12 (92.3)	0.310
	Living alone	45 (21.8)	1 (7.7)	
Residential area				0.777
	Rural	31 (15. 1)	4 (30.8)	
	Town	19 (9.2)	1 (7.7)	
	Urban	156 (75.7)	8 (61.5)	
Degree of education				0.290
	Primary school or below	35 (17.0)	4 (30.8)	
	Junior high school	29 (14. 1)	5 (38.5)	
	High school/Vocational school	121 (58.7)	4 (30.7)	
	College or above	21 (10.2)	0 (0.0)	
Monthly household income (yuan)				0.110
	<2000	42 (20.4)	6 (46. 15)	
	2000 ~ 4,999	51 (24.8)	6 (46. 15)	
	5,000 ~ 9,999	90 (43.6)	1 (7.70)	
	≥10,000	23 (11.2)	0 (0.00)	
Primary caregiver				0.753
	Spouse/Parents/Children	182 (88.4)	12 (92.3)	
	Hired helper	20 (9.7)	1 (7.7)	
	Other	4 (1.9)	0 (0.0)	

### 3.2 Analysis of disease-related characteristics of stroke patients

Among the patients, 76 used walking aids, and 77 had a history of falls within the past year. The number of stroke episodes was predominantly first-time occurrences, accounting for 36.4%. Ischemic stroke was the most common type, comprising 68.4% of cases. A total of 141 patients reported a history of smoking, and 34 reported a history of alcohol consumption. Hypertension was the most common comorbidity, affecting 91.3% of patients. The distribution of Rankin scores was as follows: 0 points (31.6%), 1 point (35.0%), 2 points (11.2%), 3 points (11.2%), 4 points (6.7%), and 5 points (4.3%). No statistically significant differences were observed in the disease-related characteristics between the 206 patients who completed the follow-up and the 13 patients lost to follow-up (*p* > 0.05). For detailed data, please refer to Table 2.

**Table 2:** Comparison of disease-related characteristics between patients who completed follow-up and those lost to follow-up (*n* (%)).

Variable	Category	Completed follow-up group (*n* = 206)	Lost-to-follow-up group (*n =* 13)	*p*
Use of walking aids	Yes	76 (36.9)	1 (7.7)	0.066
	No	130 (63. 1)	12 (92.3)	
History of falls in the past year	Yes	77 (37.4)	1 (7.7)	0.062
	No	129 (62.6)	12 (92.3)	
Number of stroke episodes				0.201
	First episode	75 (36.4)	8 (61.5)	
	2 episode	72 (35.0)	3 (23. 1)	
	>2 episode	59 (28.6)	2 (15.4)	
Type of Stroke	Hemorrhagic	65 (31.6)	1 (7.7)	0.132
	Ischemic	141 (68.4)	12 (92.3)	
Family history of stroke	Yes	43 (20.9)	2 (15.4)	1.000
	No	163 (79. 1)	11 (84.6)	
Hypertension	Yes	188 (91.3)	11 (84.6)	0.756
	No	18 (8.7)	2 (15.4)	
Diabetes	Yes	56 (27.2)	3 (23. 1)	0.968
	No	150 (72.8)	10 (76.9)	
Hyperlipidemia	Yes	48 (23.3)	3 (23. 1)	0.985
	No	158 (76.7)	10 (76.9)	
Cardiovascular disease	Yes	42 (20.4)	5 (38.5)	0.234
	No	164 (79.6)	8 (61.5)	
Kidney disease	Yes	17 (8.3)	0 (0.0)	0.606
	No	189 (91.7)	13 (100.0)	
Other diseases	Yes	7 (3.4)	0 (0.0)	0.351
	No	199 (96.6)	13 (100.0)	
Smoking history	Yes	141 (68.4)	7 (53.9)	0.432
	No	65 (31.6)	6 (46. 1)	
Alcohol history	Yes	34 (16.5)	3 (23. 1)	0.556
	No	172 (83.5)	10 (76.9)	
Rankin Score	0 No symptoms	65 (31.6)	0 (0.0)	0.183
	1 No significant disability	72 (35.0)	7 (53.9)	
	2 Slight disability	23 (11.2)	2 (15.3)	
	3 Moderate disability	23 (11.2)	3 (23. 1)	
	4 Moderately severe disability	14 (6.7)	1 (7.7)	
	5 Severe disability	9 (4.3)	0 (0.0)	

### 3.3 Scores of family resilience and self-management behaviors in stroke patients

As shown in the table, family resilience scores showed a gradual increase over time: from 80.9 ± 17.2 at T0 (hospitalization) to 99.69 ± 16.95 at T1 (3 months post-discharge), and further to 103.1 ± 16.4 at T2 (6 months post-discharge). In contrast, self-management scores first increased from 155.1 ± 27.6 at T0 to 193.2 ± 28.1 at T1, then decreased to 181.0 ± 31.1 at T2, though remaining higher than the baseline level (Table 3).

**Table 3:** Scores of family resilience and self-management behaviors in stroke patients.

Item	Maximum	Minimum	Mean ± standard deviation *(X ± S)*
T0 Family resilience	128	57	80.9 ± 17.2
T1 Family resilience	128	49	99.69 ± 16.95
T2 Family resilience	128	33	103.1 ± 16.4
T0 Self-management behaviors	239	92	155.1 ± 27.6
T1 Self-management behaviors	260	105	193.2 ± 28.1
T2 Self-management behaviors	250	97	181.0 ± 31.1

### 3.4 Common method bias test

Harman’s single-factor test was conducted by performing unrotated principal component factor analysis on the survey data from T0, T1, and T2 separately. The results showed that the variance explained by the first factor was 13.036% at T0, 16.039% at T1, and 17.214% at T2. All values were below the 40% threshold, indicating no significant common method bias in this study. However, Harman’s test alone does not fully rule out social desirability or common method bias; this limitation is discussed further.

Correlation coefficients between family resilience and self-management behaviors in stroke patients, please refer to Table 4.

**Table 4:** Correlation coefficients between family resilience and self-management behaviors in stroke patients.

Item	T0 Family resilience	T1 Family resilience	T2 Family resilience	T0 Self-management behaviors	T1 Self-management behaviors	T2 Self-management behaviors
T0 Family resilience	1.000					
T1 Family resilience	0.511^**^	1.000				
T2 Family resilience	0.354^**^	0.551^**^	1.000			
T0 Self-management behaviors	0.234^**^	0.335^**^	0.278^**^	1.000		
T1 Self-management behaviors	0.224^**^	0.349^**^	0.270^**^	0.576^**^	1.000	
T2 Self-management behaviors	0.276^**^	0.381^**^	0.180^**^	0.379^**^	0.495^**^	1.000

**p < 0.01.

### 3.5 Cross-lagged analysis of the relationship between family resilience and self-management behaviors in stroke patients

**Figure 1:**
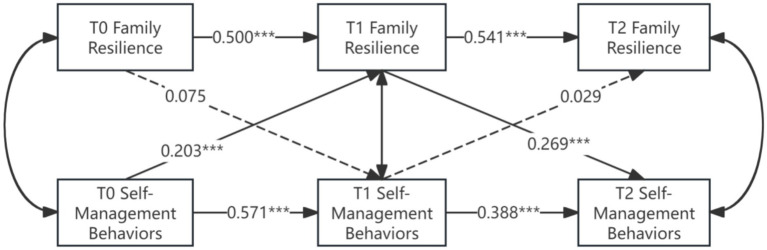
Cross-lagged analysis of family resilience and self-management behaviors. ***p < 0.001, **p < 0.01, *p < 0.05; Dashed paths are non-significant.

A cross-lagged model was constructed to examine the relationship between family resilience and self-management behaviors. The model demonstrated excellent fit to the data: χ^2^/df = 0.927, CFI = 1.000, TLI = 1.004, RMSEA = 0, SRMR = 0.019. Given the low degrees of freedom (df = 4), the nearly perfect fit indices (CFI = 1.000, RMSEA = 0.000) are largely a consequence of model simplicity; therefore, our conclusions rely primarily on the significance and direction of path coefficients rather than on absolute fit indices. The results are presented in Figure 1.

## 4 Discussion

### 4.1 Stroke patients’ self-management behaviors initially increased, then decreased over time

This study revealed that the self-management behaviors of stroke patients followed an initial increase followed by a subsequent decline over time, demonstrating a significant time effect (*p* < 0.05) with no notable gender differences, which aligns with the findings of Zhou (2024). Specifically, the total self-management behavior score at T0 (during hospitalization) was 155.14 ± 27.62, indicating a moderately low level. Further analysis showed that most patients and their caregivers had insufficient understanding of systematic and scientific self-management, with particularly low scores in the dimensions of disease management, medication safety management, and daily living management. This may be attributed to two possible reasons: first, patients’ inadequate knowledge about the disease itself, as knowledge level has been confirmed as a key influencing factor for self-management (Qin, 2020); second, during hospitalization, patients tend to develop excessive reliance on medical staff, exhibit low personal initiative, and their initial anxiety may further impair rehabilitation motivation (Forster et al., 2013).

Longitudinal data indicated a significant increase in patients’ self-management behavior levels from hospitalization to 3 months post-discharge, with the total score rising from 155.14 ± 27.62 to 193.2 ± 28.1. Improvements in daily living management and rehabilitation exercise management were particularly pronounced. This phase of improvement is consistent with studies by Yang (2022) and Wei (2018), mainly attributable to a significant enhancement in health awareness and maintained high exercise adherence and rehabilitation willingness within the first 3 months after stroke (Damush et al., 2007).

However, from 3 to 6 months post-discharge, the level of self-management behaviors showed a clear decline, dropping from 193.2 ± 28.1 to 181.0 ± 31.1, although it did not fall to the initial level. This observation is consistent with that of Wang (2022). The decline in behavior may be multifaceted: the heightened vigilance present in the early stages of the illness diminishes over time, potentially leading to reduced self-efficacy and decreased adherence to health behaviors (Jones and Riazi, 2011). Concurrently, post-stroke fatigue may not only deplete the energy required for patients to perform self-management but also diminishes their social participation (Wang et al., 2025), collectively contributing to the decline in behavioral levels.

This trajectory suggests that patients’ self-management behaviors fail to be sustained long-term, and reliance on policies and initial health education is insufficient to ensure long-term outcomes. Therefore, based on the support system, it is imperative to establish a collaborative management network involving the clinical team and the family, spanning different recovery stages, and emphasizing the synergistic role of clinical healthcare professionals and the family. Clinical nurses should assume the role in transitional care, providing individualized education starting during hospitalization, with a focus on guiding medication safety, daily living, and rehabilitation exercises. After discharge, they should continue supervision through follow-ups and remote support, strengthening reminders and motivation around 3 months post-discharge when behavior is prone to decline. Doctors need to reinforce health education throughout the treatment process, collaboratively develop rehabilitation plans before discharge, and regularly assess and adjust management plans during follow-up visits. Family members should act as companions and assistants, learning care knowledge, providing emotional support, behavioral supervision, and environmental facilitation, helping patients overcome the challenges of post-stroke fatigue and declining motivation. Through a model combining professional medical support and family cooperation, and by constructing an integrated in-hospital and post-discharge care network, the declining trend in patients’ self-management behaviors can be effectively delayed, thereby enhancing long-term quality.

### 4.2 Family resilience in stroke patients showed an initial rapid increase followed by a slower rise

Repeated-measures ANOVA revealed that family resilience among stroke patients changed significantly over time (*F* (1.89, 387.67) = 196.00, *p* < 0.001, partial η^2^ = 0.489). Descriptive data showed that the total score increased from 80.9 ± 17.2 during hospitalization to 103.1 ± 16.4 at 6 months post-discharge. Further analysis indicated that the increase in family resilience followed a pattern of “rapid initial growth followed by a slowdown”: a swift increase from hospitalization to 3 months post-discharge (an approximate gain of 18.6 points), followed by a markedly slower rate of increase that tended to stabilize from 3 to 6 months post-discharge (an approximate gain of 3.3 points). This trajectory is consistent with the findings of Zhang et al. (2022).

The underlying reasons are as follows. In the initial stage of the illness (from hospitalization to 3 months post-discharge), families rapidly mobilized internal and external resources and established new coping patterns to manage the crisis. The enhancement in the dimension of social resource utilization was particularly crucial, serving not only as a primary source of resilience growth but also strengthening bonds among family members. However, as time progressed, the challenges faced by families shifted from an acute crisis to chronic issues such as long-term care pressure, economic burden, and the patient’s social participation. Coupled with potential adaptation fatigue and resource depletion following the initial high-stress state, these factors collectively contributed to the deceleration in the growth rate of family resilience during the later period.

Furthermore, the level of family resilience observed during hospitalization in this study was lower than that reported in studies by Zhao et al. (2024) and Teng et al. (2023). This discrepancy may point to the influence of healthcare resource accessibility. In regions with abundant resources, families can more easily access professional support, thereby enhancing their coping capacity. Conversely, in resource-scarce settings, families must bear more pressure independently, which may constrain the initial development of their resilience.

This changing trajectory suggests that during the initial period of rapid resilience growth (hospitalization and early post-discharge), healthcare professionals should accurately assess the level of social support available to the patient’s family. They should proactively guide and assist families in effectively integrating and utilizing various social resources. As the illness enters a later stage with slowed resilience growth (after 3 months post-discharge), healthcare professionals should conduct regular follow-ups to assess potential issues such as caregiver burnout, economic pressure, and social isolation within the family. They should provide sustained psychological support, caregiving skills guidance, and encourage the family to rebuild their social life. On this basis, medical institutions should strengthen collaboration with community hospitals and rehabilitation centers to establish an integrated “hospital-community-family” rehabilitation model, laying the foundation for the patient’s holistic recovery process.

### 4.3 Interaction between family resilience and self-management behaviors in stroke patients

Cross-lagged analysis revealed a dynamic, time-evolving interactive mechanism between family resilience and self-management behaviors in stroke patients:

1. Self-management at T0 predicted family resilience at T1 (*β* = 0.203, 95% CI [0.098, 0.307], *p* < 0.001), while family resilience at T0 did not significantly predict self-management at T1. This indicates that early self-management is an important predictor of family resilience. Patients’ active behaviors (e.g., timely medication, symptom monitoring, healthy lifestyle) may reflect regained control and reduce family caregiving burden and psychological pressure (Grey et al., 2006). Secondly, successful self-management may enhance the patient’s confidence and sense of efficacy (Lynch et al., 2025), making them more willing to engage in positive communication with family members and jointly solve problems, thereby becoming a positive force within the family system and reducing the family caregiving burden (Banitalebi et al., 2022). Moreover, the patient’s behavior provides a positive model and hope for other family members, motivating the whole family to adopt more flexible coping strategies.
2. Family resilience at T1 predicted self-management at T2 (*β* = 0.269, 95% CI [0.136, 0.444], *p* < 0.001), whereas self-management at T1 did not significantly predict family resilience at T2. Thus, over time, family resilience becomes a key predictor of self-management. Higher-resilience families provide stable emotional support, effective resource mobilization, and a positive communication environment (Zhang et al., 2024), which collectively may help patients maintain good self-management behaviors. When the family system possesses stronger adaptation and recovery capabilities, patients may find it easier to sustain adherence and initiative in long-term disease management (Rosland and Piette, 2010), forming a virtuous cycle.
3. The non-significant path from self-management at T1 to family resilience at T2 (*β* = 0.089, *p* = 0.214) suggests that the predictive effect of self-management on family resilience may be time-limited. One possible explanation is that by three months post-discharge, families have already adapted to the caregiving role and established stable coping patterns, leaving less room for additional influence from patients’ self-management behaviors. Alternatively, the sustained burden of caregiving from 3 to 6 months may overshadow the positive effects of patient self-management, preventing further enhancement of family resilience during this period.

### 4.4 Limitations

This study has several limitations. First, data collection relied on patient self-reports, which may be subject to social desirability bias or recall bias. Although Harman’s test suggested no severe common method bias, this approach does not fully eliminate concerns regarding self-report biases. Test–retest reliability for the FRAS-C and SSBS was not assessed in this study, which is acknowledged as a limitation. Second, although some factors were controlled for, there may still be other unmeasured confounding factors (such as specific family financial pressure and social support) that could provide additional explanatory power for the relationship between family resilience and self-management behaviors. The cross-lagged model did not include covariates such as stroke severity, comorbidities, or socioeconomic status, which may influence both family resilience and self-management. Third, the follow-up duration was limited. This study only tracked patients until 6 months after discharge, whereas stroke rehabilitation is a long-term process. The short follow-up period may not fully capture the long-term dynamic trajectories and subsequent patterns of the relationship between the two variables. Fourth, the convenience sample from only two hospitals in a single city limits the generalizability of the findings. The observed relationships may be influenced by cultural and healthcare system factors specific to China, such as strong family involvement in care and limited community rehabilitation resources. Future research should extend the follow-up period and incorporate a broader range of potential factors to generate more robust findings.

## 5 Conclusion

Stroke patients’ self-management behaviors show an “initial increase followed by decline” over six months post-discharge, while family resilience shows a “rapid initial increase then slower rise.” Cross-lagged analysis reveals a time-ordered bidirectional relationship: early self-management (hospitalization to 3 months) positively predicts later family resilience, and subsequent family resilience (3–6 months) predicts sustained self-management. Thus, early rehabilitation should focus on building patients’ self-management skills, whereas mid-to-late rehabilitation should strengthen family resilience resources. An integrated “hospital-community-family” model targeting these critical windows can promote a virtuous patient-family interaction cycle and improve long-term outcomes.

## Data Availability

The original contributions presented in the study are included in the article/supplementary material, further inquiries can be directed to the corresponding authors.
